# Supplementary material for: The Impact of Diet and Physical Activity on Fat-to-Lean Mass Ratio
Source: Nutrients. 2023 Dec 20;16(1):19. doi: 10.3390/nu16010019 (PMC10780510; doi:10.3390/nu16010019)
Supplement: Supplementary file 1 [file nutrients-16-00019-s001.zip › nutrients-2745457_Supplementary File S1_ Sample Low-Calorie Mediterranean Diet Meal Plan.pdf]

Supplementary File S1: Sample Low-Calorie Mediterranean Diet Meal Plan

This appendix provides an example of a typical day's meal plan under the low-calorie Mediterranean Diet used in our study. The plan is nutritionally balanced, adhering to the Mediterranean diet principles while maintaining a suitable caloric content for weight management.

Total Daily Caloric Intake: Approximately 1500-1700 Kcal

Breakfast
White milk yogurt: 120 g
Whole grain bread: 60 g
Fresh fruit juice (two fruits + vegetables): 150 g
Caloric Content: ~300-350 Kcal

Mid-Morning Snack
Fresh fruit (e.g., apple, pear): 150 g
Nuts (low- salt): 10 g
Caloric Content: ~150-200 Kcal

Lunch
Brown rice: 60 g
Legumes : 80 g or Grilled fish (e.g., Anchovies): 80 g
Freshly cooked vegetables (e.g., steamed broccoli, carrots): 300 g
"Extra virgin" olive oil 15g
Caloric Content: ~450-500 Kcal

Afternoon Snack
Fresh fruit (e.g., orange): 150 g
Soy or 2% Greek yogurt: 120 g
Caloric Content: ~150-200 Kcal

Dinner
Whole grain pasta: 80 g
Freshly cooked vegetables (e.g., steamed broccoli, carrots): 300 g
"Extra virgin" olive oil 15g
Caloric Content: ~300-350 Kcal

Note: This meal plan is a guideline and can be adjusted according to individual dietary needs and preferences. Portion sizes and ingredients can be varied to meet specific calorie targets and nutritional requirements. All weights mentioned are for raw ingredients.
