# Supplementary material for: The Impact of Diet and Physical Activity on Fat-to-Lean Mass Ratio
Source: Nutrients. 2023 Dec 20;16(1):19. doi: 10.3390/nu16010019 (PMC10780510; doi:10.3390/nu16010019)
Supplement: Supplementary file 1 [file nutrients-16-00019-s001.zip › nutrients-2745457_Table 1S - Body composition of all subjects before and after the two months study.pdf]

Table 1S. - Body composition of all subjects before and after the two months' study.

Variable	T0	T1	Δ (T1-T0)	p
**Whole Sample**				
WEIGHT (kg)	82.7 ± 20.0	79.7 ± 19.3	-2.9 ± 1.8	<0.001
BMI (Kg/m2)	28.7 ± 5.4	27.7 ± 5.3	-1.0 ± 0.6	<0.001
FM (kg)	25.9 ± 10.5	23.5 ± 9.8	-2.4 ± 1.7	<0.001
FM (%)	30.7 ± 7.8	28.9 ± 7.9	-1.8 ± 1.5	<0.001
FFM (kg)	54 ± 12.9	53.5 ± 12.9	-0.5 ± 1.4	0.0012
FM-to-FFM ratio	0.49 ± 0.18	0.45 ± 0.18	-0.04 ± 0.03	<0.001
TBW (kg)	40.4 ± 9.7	39.8 ± 9.5	-0.6 ± 1.0	<0.001
Body Protein (Kg)	13.4 ± 3.6	13.7 ± 3.5	0.3 ± 1.0	0.0049
**Females**				
WEIGHT (kg)	73.1 ± 14.5	70.6 ± 13.9	-2.5 ± 1.8	<0.001
BMI (Kg/m2)	27.2 ± 5.1	26.3 ± 4.9	-0.9 ± 0.6	<0.001
FM (kg)	25.4 ± 10.4	23.4 ± 10.0	-2.1 ± 1.4	<0.001
FM (%)	33.6 ± 6.9	31.9 ± 7.3	-1.7 ± 1.4	<0.001
FFM (kg)	45.3 ± 5.3	45.0 ± 5.2	-0.4 ± 1.2	0.0270
FM-to-FFM ratio	0.55± 0.18	0.51 ± 0.18	-0.04 ± 0.03	<0.001
TBW (kg)	33.8 ± 4.4	33.5 ± 4.2	-0.4 ± 1.0	0.0079
Body Protein (Kg)	11.1 ± 1.4	11.5 ± 1.2	0.4 ± 1.0	0.0079
**Males**				
WEIGHT (kg)	97.0 ± 18.6	93.5 ± 18.1	-3.5 ± 1.7	<0.001
BMI (Kg/m2)	31.0 ± 5.2	29.9 ± 5.0	-1.1 ± 0.5	<0.001
FM (kg)	26.5 ± 10.8	23.6 ± 9.8	-2.8 ± 2.0	<0.001
FM (%)	26.3 ± 7.1	24.4 ± 6.7	-1.9 ± 1.6	<0.001
FFM (kg)	67.1 ± 9.5	66.5 ± 9.8	-0.7 ± 1.7	0.0190
FM-to-FFM ratio	0.39 ± 0.14	0.35 ± 0.13	-0.04 ± 0.04	<0.001
TBW (kg)	50.2 ± 6.8	49.4 ± 7.0	-0.8 ± 0.9	<0.001

Body Protein (Kg)	16.9 ± 3.2	17.1 ± 3.2	0.2 ± 1.0	0.2496
**BMI 18.5-24.9**				
WEIGHT (kg)	63.2 ± 6.7	61.1 ± 6.6	-2.1 ± 1.5	<0.001
BMI (Kg/m2)	23.0 ± 1.5	22.2 ± 1.4	-0.8 ± 0.6	<0.001
FM (kg)	15.8 ± 4.2	14.1 ± 4.0	-1.7 ± 1.1	<0.001
FM (%)	25.1 ± 6.2	23.1 ± 5.8	-2.0 ± 1.5	<0.001
FFM (kg)	45.1 ± 6.8	44.8 ± 6.4	-0.4 ± 1.0	0.0598
FM-to-FFM ratio	0.37 ± 0.12	0.33 ± 0.11	-0.04 ± 0.03	<0.001
TBW (kg)	33.3 ± 5.2	33.0 ± 4.8	-0.4 ± 0.8	0.0230
Body Protein (Kg)	11.7 ± 1.9	11.8 ± 1.7	0.1 ± 0.7	0.4847
**BMI 25-29.9**				
WEIGHT (kg)	77.3 ± 10.0	74.7 ± 9.8	-2.5 ± 1.2	<0.001
BMI (Kg/m2)	27.5 ± 1.5	26.6 ± 1.4	-0.9 ± 0.4	<0.001
FM (kg)	24.1 ± 4.8	22.1 ± 4.7	-2.0 ± 0.9	<0.001
FM (%)	31.5 ± 6.3	29.9 ± 6.5	-1.6 ± 1.0	<0.001
FFM (kg)	50.5 ± 9.9	50.0 ± 9.9	-0.5 ± 1.1	0.0087
FM-to-FFM ratio	0.49 ± 0.13	0.45 ± 0.13	-0.04 ± 0.03	<0.001
TBW (kg)	37.5 ± 7.1	37.2 ± 7.1	-0.4 ± 0.8	0.0094
Body Protein (Kg)	12.7 ± 3.2	12.8 ± 3.0	0.2 ± 0.8	0.1191
**BMI 30-34.9**				
WEIGHT (kg)	93.6 ± 8.5	90.0 ± 8.6	-3.6 ± 1.6	<0.001
BMI (Kg/m2)	31.7 ± 1.3	30.5 ± 1.5	-1.2 ± 0.6	<0.001
FM (kg)	29.8 ± 5.5	26.9 ± 5.5	-2.9 ± 2.0	<0.001
FM (%)	32.0 ± 6.5	30.1 ± 6.5	-1.9 ± 2.0	0.0010
FFM (kg)	60.6 ± 9.2	60.0 ± 8.7	-0.6 ± 1.8	0.1724
FM-to-FFM ratio	0.61 ± 0.19	0.56 ± 0.17	-0.05 ± 0.05	0.001
TBW (kg)	45.6 ± 6.4	44.8 ± 6.2	-0.9 ± 1.1	0.0030
Body Protein (Kg)	15.0 ± 3.2	15.3 ± 2.8	0.3 ± 1.1	0.3148

**Age 18-29**				
WEIGHT (kg)	77.6 ± 13.4	74.7 ± 12.6	-2.9 ± 1.3	<0.001
BMI (Kg/m2)	26.1 ± 2.4	25.7 ± 3.2	-1 ± 0.4	<0.001
FM (kg)	21.9 ± 7.7	19.8 ± 7.3	-2.1 ± 1.0	<0.001

FM (%)	28.1 ± 8.2	26.4 ± 8.1	-1.8 ± 1.1	<0.001
FFM (kg)	53.0 ± 10.9	52.3 ± 10.4	-0.7 ± 1.0	0.001
FM-to-FFM ratio	0.43 ± 0.17	0.39 ± 0.16	-0.04 ± 0.02	<0.001
TBW (kg)	39.6 ± 7.6	39.1 ± 7.5	-0.5 ± 1.0	0.001
Body Protein (Kg)	13.2 ± 3.4	13.2 ± 3.2	-0.01 ± 0.6	0.928
**Age 30-49**				
WEIGHT (kg)	84.5 ± 29	81.5 ± 21.7	-3.0 ± 2.0	<0.001
BMI (Kg/m2)	29 ± 5.7	28 ± 5.5	-1.0 ± 0.7	<0.001
FM (kg)	26.3 ± 10.4	23.7 ± 9.4	-2.6 ± 1.9	<0.001
FM (%)	30.6 ± 6.9	28.6 ± 6.9	-2.1 ± 1.6	<0.001
FFM (kg)	55.3 ± 14.4	55.0 ± 14.4	-0.3 ± 1.6	0.226
FM-to-FFM ratio	0.48 ± 0.16	0.43 ± 0.15	-0.05 ± 0.03	<0.001
TBW (kg)	41.2 ±10.9	40.7 ± 10.7	-0.5 ± 1.0	0.009
Body Protein (Kg)	13.9 ± 3.9	14.3 ± 3.8	0.4 ± 1.1	0.0111
**Age 50-65**				
WEIGHT (kg)	86.7 ± 20.1	84.05 ± 19.55	-2.6 ± 1.9	0.001
BMI (Kg/m2)	32.8 ± 6.3	31.79 ± 6.22	-1.0 ± 0.7	0.0009
FM (kg)	33.9 ± 13.1	32.38 ± 12.90	-1.6 ± 1.7	0.0124
FM (%)	38.1 ± 7.2	37.44 ± 7.21	-0.7 ± 1.3	0.1148
FFM (kg)	50.1 ± 9.3	48.91 ± 8.95	-1.2 ± 1.6	0.0312
FM-to-FFM ratio	0.67 ± 0.22	0.66 ± 0.22	-0.02 ± 0.04	0.2184
TBW (kg)	37.9 ± 8	36.9 ± 7.7	-1.0 ± 1.0	0.0107
Body Protein (Kg)	11.4 ± 2.3	12.0 ± 1.6	0.6 ± 1.0	0.0927

This table presents the mean ± SD for each variable at baseline (T0) and follow-up (T1), along with the change (Δ) and p-value. It includes data for the whole sample and subgroups based on BMI and age. Variables include weight, BMI, fat mass (FM), fat-free mass (FFM), total body water (TBW), and body protein. n: number of subjects. Abbreviations: T0 = Baseline, T1 = Follow-up, Δ = Change from T0 to T1, p = p-value. Independent sample t-tests were used to determine the statistical significance for the P-values presented.
